# Supplementary figures and images for: Estrogen Mediates the Sexual Dimorphism of GT1b-Induced Central Pain Sensitization
Source: Cells. 2023 Mar 6;12(5):808. doi: 10.3390/cells12050808 (PMC10001026; doi:10.3390/cells12050808)

**A**Log<sub>2</sub> (fold change)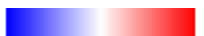

-3      0      3

**M-GT1b/M-Veh****F-GT1b/F-Veh**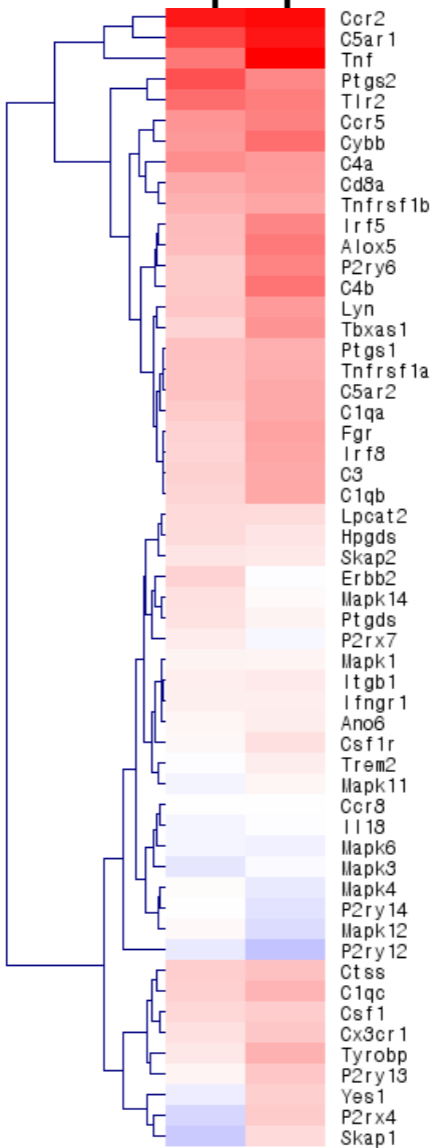

Supplement: Supplementary file 1 [file cells-12-00808-s001.zip › cells-2204528-supplementary/Supplementary Figure S1. Sex-dependent transcriptional regulation of pain-related genes_V2.pdf]

A

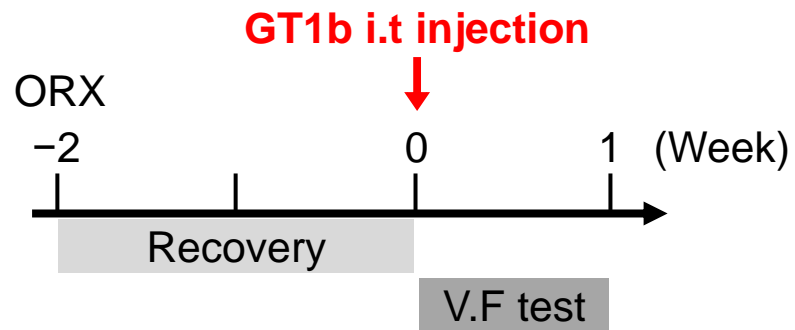

B

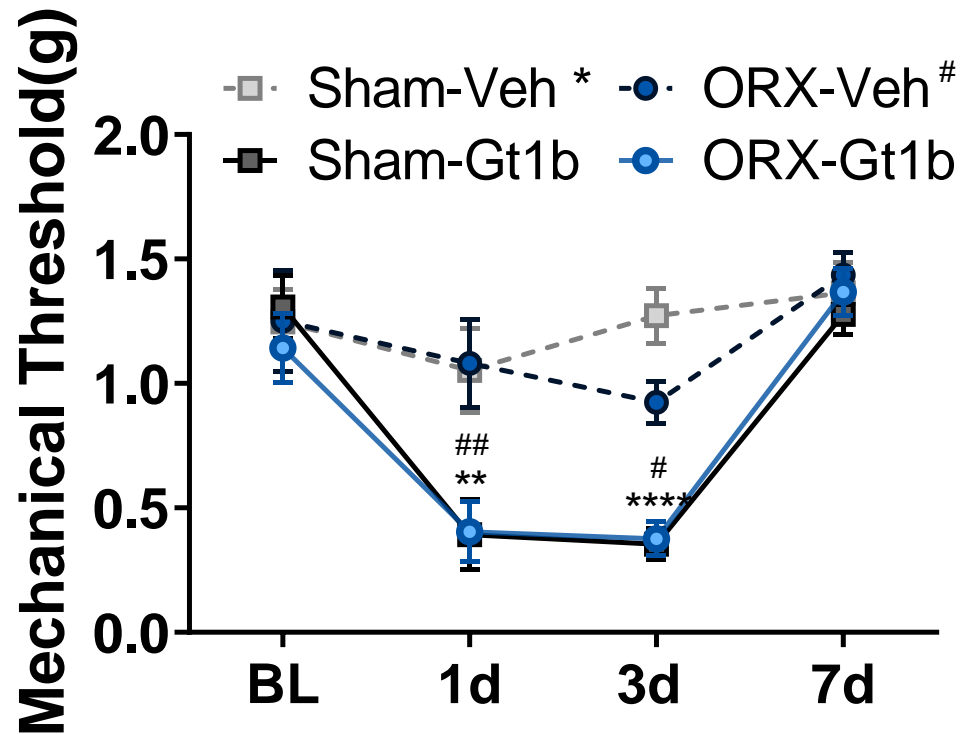

Supplement: Supplementary file 1 [file cells-12-00808-s001.zip › cells-2204528-supplementary/Supplementary Figure S2. GT1b-induced pain centralsensitization in orchiectomized male.pdf]
